# Identification of Sex-Associated Genetic Markers in *Pistacia lentiscus* var. *chia* for Early Male Detection

**DOI:** 10.3390/genes15050632

**Published:** 2024-05-16

**Authors:** Evangelia Stavridou, Ioanna Karamichali, Evangelos Siskas, Irini Bosmali, Maslin Osanthanunkul, Panagiotis Madesis

**Affiliations:** 1 Department of Botany, School of Biology, Aristotle University of Thessaloniki, 54124 Thessaloniki, Greece; estavrido@bio.auth.gr (E.S.); siskasen@bio.auth.gr (E.S.); 2 Laboratory of Agrobiotechnology and Molecular Plant Breeding, Institute of Applied Biosciences (INAB), Center for Research and Technology (CERTH), 57001 Thessaloniki, Greece; ikaramichali@certh.gr (I.K.); eirinimposmali@certh.gr (I.B.); 3 Department of Biology, Faculty of Science, Chiang Mai University, Chiang Mai 50200, Thailand; maslin.o@cmu.ac.th; 4 Research Centre in Bioresources for Agriculture, Industry and Medicine, Chiang Mai University, Chiang Mai 50200, Thailand; 5 Laboratory of Molecular Biology of Plants, Department of Agriculture Crop Production and Rural Environment, School of Agricultural Sciences, University of Thessaly, 38446 Volos, Greece

**Keywords:** sex identification, mastic tree, RAPD, ISSR, SCAR, EST-SSR, SNPs

## Abstract

*Pistacia lentiscus* var. *chia* is a valuable crop for its high-added-value mastic, a resin with proven pharmaceutical and cosmeceutical properties harvested from the male tree trunk. To achieve the maximum economic benefits from the cultivation of male mastic trees, it is important to develop early sex diagnosis molecular tools for distinguishing the sex type. Thus far, the work on sex identification has focused on *Pistacia vera* with promising results; however, the low transferability rates of these markers in *P. lentiscus* necessitates the development of species-specific sex-linked markers for *P. lentiscus* var. *chia*. To our knowledge, this is the first report regarding: (i) the development of species-specific novel transcriptome-based markers for *P. lentiscus* var. *chia* and their assessment on male, female and monoecious individuals using PCR-HRM analysis, thus, introducing a cost-effective method for sex identification with high accuracy that can be applied with minimum infrastructure, (ii) the effective sex identification in mastic tree using a combination of different sex-linked ISSR and SCAR markers with 100% accuracy, and (iii) the impact evaluation of sex type on the genetic diversity of different *P. lentiscus* var. *chia* cultivars. The results of this study are expected to provide species-specific markers for accurate sex identification that could contribute to the selection process of male mastic trees at an early stage for mass propagation systems and to facilitate future breeding efforts related to sex-linked productivity and quality of mastic resin.

## 1. Introduction

Sex identification in dioecious plants, at an early developmental pre-flowering stage, is challenging; yet of great economical and biological importance, especially for species producing high-added-value products, such as the mastic tree (*Pistachia lentiscus* L. var. *chia*). *P. lentiscus* L. is widely distributed in the maquis communities of the Mediterranean basin and the economically important variety *chia* is grown in the island of Chios in the northern Aegean Sea. Mastic gum or Chios mastiha, is the resinous sap extracted from the tree trunk and the main branches [1] as a reaction of the of the male mastic trees to wounding. Chios mastiha, which is known for its therapeutic properties, has been identified as a Protected Designation of Origin (PDO) product by the European Commission, 1997 [2] and in 2014 the know-how of cultivating mastic trees in Chios was inscribed in the Representative List of the Intangible Cultural Heritage of Humanity (UNESCO), 2014 [3].

Substantial advancements have been made mainly in *Pistacia vera*, using conventional breeding and selection methods. However, there is limited scientific research on Pistacia wild species and specifically on *P. lentiscus* [4], albeit the great economic importance of *P. lentiscus* var. *chia* for production of the mastic gum, which is typically propagated vegetatively by hardwood shoot cuttings [5] and breeding efforts are still in their infancy. Two major limiting factors of breeding capacity in the perennial *Pistacia* species are the required long breeding cycles, and their dioecious nature, which entail additional costs, labor, time, and land for preserving and testing the breeding populations [6,7]. The use of various DNA-based molecular markers developed for *P. vera* for the study of intra- and interspecific phylogeny, and marker-assisted breeding have only recently begun to be applied in *P. lentiscus* mainly for male cultivar fingerprinting, genotype identification, and genetic diversity analysis in wild genotypes of *P. lentiscus*.

A major breeding target in *P. lentiscus* var. *chia* would be the maximum productivity of male trees for high-quality mastic gum. The male mastic trees can reach full sexual maturity after 5–6 years, whilst mastic production reaches a maximum yield of 1 kg at 12–15 years [8]. As such, it is essential for the mastic tree breeders, growers, and producers to distinguish the male trees in early growth stages. Therefore, early-stage sex identification is crucial given the importance of distinguishing the male seedlings in mass propagation systems, which produce higher quantities and better-quality of mastic compared to the female trees. Thus far, sex identification in *Pistacia* species is unattainable during the long pre-reproductive phase of the trees, due to the lack of sex-related morphological methods at this developmental stage [9,10,11]. The application of appropriate species-specific molecular markers could be an effective molecular tool and facilitate sex identification in mastic trees at the pre-reproductive phase.

Research on the sex determination mechanism in *P. vera* L. has shown that the development of opposite sex primordia is initiated but then arrested in the early stages [12]. Based on sex-associated loci identified in pistachio, a ZZ/ZW sex determination system has been reported, in which the females are the heterogametic sex [6,10,13,14]. Furthermore, there is evidence that the heterogametic sex chromosome system is emerging in the early stage of differentiation given that the heterozygous SNPs markers are present in pistachio females [10]. Interestingly and despite the dioecious nature of *Pistacia* species, monoecious instances with either male and female inflorescences on different branches, or on the same branch or mixed inflorescences and bisexual flowers, have also been previously observed in wild species [15], such as in *Pistacia chinensis* Bunge [16], *Pistacia atlantica* Desf. [17,18], and *Pistacia terebinthus* [19]. Similar cases have also been observed in *P. lentiscus* L. trees on the island of Chios (Chios Mastic Gum Growers Association).

Sex identification in *Pistacia* species has largely focused in *Pistacia vera* using various sex-linked DNA markers, such as different Random Amplified Polymorphic DNA (RAPD) markers in *P. vera* [9,20,21], *Pistacia eurycarpa* and *P. atlantica* [17]. More specifically, the OPO-08 decameric Operon primer (RAPD), successfully distinguished the female individuals in *P. vera,* resulting in a 945 bp amplification product [20]. However, in other instances, sex discrimination with this RAPD marker was not possible in *P. vera* [22]. In a later study, Yakubov et al. [23] transformed the OPO-08 RAPD primer into Sequence Characterized Amplified Region (SCAR) marker that enabled the discrimination between male and female individuals in different *P. vera* genotypes [23] and in other *Pistacia* species [9,21]. Based on these studies, the low frequency of sex-linked bands observed indicates that the genetic loci involved in sex determination are small and probably involve a single or very few genes [9,20]. Testing the SCAR marker in *P. vera* gave false-negative and false-positive results in females and males, respectively, and was not effective in distinguishing sex in other *Pistacia* species [7]. Effective sex identification of female and male individual *P. vera* cultivars has been also achieved using Inter-Simple Sequence Repeat (ISSR) markers [24]. Sex-linked polymorphic EST-SSR markers were also effective in eliminating males in *P. vera* yet were not able to successfully identify the sex in other Pistacia species [6], except for the transcriptome-based EST-SSR marker identified by Chang et al. [25], which was effective in distinguishing between male and female *P. chinensis*.

The development of species-specific markers for sex identification in *P. lentiscus* var. *chia* is still lacking, especially at the juvenile period. The use of ISSR and RAPD markers for genetic diversity studies in *P. lentiscus* L. have also shown a great potential for sex identification, whereas SSRs were not as effective in distinguishing male from female individuals [26]. Specifically, there is evidence that male and female plants were distinguished using RAPD and ISSR markers in *P. lentiscus* L. (12 females vs. 12 males) [26] and *P. lentiscus* L. var. *chia* genotypes, (one female vs. 10 males) [27]; 10 females vs. 10 males) [28], however these studies used only a limited number of male and female individuals, thus further work is required to be able to confidently differentiate male from female individuals in *P. lentiscus* and especially in the variety *chia*.

To achieve the maximum economic benefits from the cultivation of *P. lentiscus* var. *chia*, it is necessary to distinguish males from females at the seedling stage through early sex diagnosis tools. Herein, genetic markers such as ISSRs, RAPDs and SCARs, which have shown promising results in other closely related Pistacia species, were used to assess the efficacy in sex differentiation between female and male mastic trees. These markers were further used to assess potential links between the sex identity and the genetic diversity of different mastic tree cultivars. Additionally, transcriptome-based markers were developed as an alternative for early-stage sex diagnosis in *P. lentiscus* var. *chia* using male, female, and monoecious individuals. The results of this study are promising as a desirable tool for accelerating the selection process for male seedlings at an early stage and reducing the costs of and time required in breeding programs.

## 2. Materials and Methods

### 2.1. Plant Material

Plant material consisted of leaves collected from 44 productive males, 5 non-productive males, 20 females and 5 monoecious mature mastic trees from fields in southern Chios. For the sex identification, two approaches were followed using: (i) sex-linked ISSR, RAPD and SCAR markers based on literature and (ii) transcriptome-based developed primers. The total DNA was isolated from approximately 0.2 g of leaf material for each sample following a modified version of the Cetyl Trimethyl Ammonium Bromide (CTAB) protocol [29]. DNA concentration and quality were determined using the UV-Vis Spectrophotometer Q5000 (Quawell Technology Inc., San Jose, CA, USA). Samples were then diluted to 10 ng µL^−1^. The molecular markers used for the sex identification in mastic trees are presented in Table 1.

### 2.2. PCR Amplification

Extracted genomic DNA was PCR-amplified using 14 ISSRs, 2 SCARs and 1 RAPD markers (Table 1). Polymerase chain reactions (PCR) were performed in a SureCycler 8800 thermal cycler (Agilent Technologies, Santa Clara, CA, USA). The PCR reactions for the different markers were carried out in a final volume of 25 μL and the reaction mixture was prepared as follows: 30 ng of genomic DNA, 1X Reaction Buffer, 2 mM MgCl_2_, 0.2 mM dNTPs, 1U KAPA Taq polymerase (Kapa Biosystems, Inc., Wilmington, MA, USA) and primer concentration was as follows: 2 μΜ for the (AC)_8_CG and (AC)_8_TA, 0.8 μM for the ISSR, 0.5 μM for the OPO-08, 0.4 μM for the PVF1-PVF2, and 0.2 μM for the SCO-08. The cycling profile after several trials for effective amplification was carried out as follows: for the ISSR markers, an initial denaturation step at 94 °C for 4 min was followed by 35 cycles of 30 sec denaturation at 94 °C, annealing at the respective Ta for each primer (Table 1) for 40 s and extension at 72 °C for 50 s. For the OPO-08, an initial denaturation step at 94 °C for 2 min was followed by 45 cycles of 45 s denaturation at 94 °C, annealing at 32 °C for 1 min and extension at 72 °C for 2 min. For the SCO-08 marker, denaturation was performed at 94 °C for 3 min followed by 30 cycles of 1 min denaturation at 94 °C, annealing at 56 °C for 1 min and extension at 72 °C for 2 min. Respectively, for the touchdown PCR of the PVF1-PVF2 marker, denaturation was performed at 94 °C for 5 min followed by 20 cycles of denaturation at 94 °C for 30 s, annealing at 60–50 °C for 30 s (0.5 °C per cycle) and extension at 72 °C for 1 min. Then a second step with 20 cycles of denaturation at 94 °C for 30 s, annealing at 51.2 °C for 30 s and extension at 72 °C for 30 s. All PCRs were followed by a final extension step at 72 °C for 5 min. The PCR products were then separated by electrophoresis in 2% agarose gel with 1X TAE stained with ethidium bromide and the use of Quick-Load^®^ 1Kb plus DNA ladder (New England Biolabs, Ipswich, MA, USA). Gel images were visualized with the UV Minibis Pro (DNR Bio-Imaging Systems, Jerusalem, Israel) instrument and the scoring was performed using the Logger Pro 3.15 software.

### 2.3. Transcriptome-Based Marker Design

For the development of molecular markers, literature-based sequences of known genes that are important for sex determination [6,15,30,31,32] and flowering in plants [33,34,35,36] were extracted from KEGG, wikipathways (https://www.wikipathways.org/index.php/Pathway:WP2312, accessed on 25 May 2021), and NCBI and databases available on mpipz.mpg.de (https://www.mpipz.mpg.de/14637/Arabidopsis_flowering_genes, accessed on 25 May 2021). The reference sequences were used as queries with Blastn (v. 2.6.0+) for the homologous gene search in two datasets, including the dataset of differentially expressed genes and the dataset of the EST-SSR sequences. These datasets were developed for each sex group (male and female) using the draft transcriptome of the mastic tree, which was assembled using Trinity (v. 2.8.5) and the raw reads released in the NCBI Sequence Read Archive (SRA) database under the BioProject accession number PRJNA918300. The differentially expressed genes in male and female trees were identified by mapping the reads on the draft transcriptome with the program Salmon (v. 0.14.1) and by analysing the gene counts with the R (v. 4.0.2) package DESeq2 (v. 1.28.1). SNPs/Indels, and EST-SSR sequence datasets developed for each sex group (male and female). In parallel, EST-SSR sequence datasets were developed for each sex group (male and female), using the program MISA (v. 2.1). The homologous to the genes of reference sequences for each sex group were extracted, sex specific SNPs and Indels were identified and further used for the development of potential genetic markers for early-stage sex identification.

### 2.4. RT-PCR Assessment of the EST-SSR and DEG-Based Markers Coupled with HRM

To test whether the transcriptome-based markers were effective in identifying the different sex in *P. lentiscus* var. *chia*, 34 male and 20 female individuals were used. The PCR reaction mixtures were prepared in a final volume of 20 μL containing 1X KAPA Taq Buffer (Kapa Biosystems, Wilmington, MA, USA), 0.2 mM dNTPs, 0.6 mM of each primer, 1.5mM Syto^®^ 9 green fluorescent nucleic acid stain, 1 U Kapa Taq DNA polymerase and 40 ng of the DNA template. The PCR amplification, High Resolution Melting (HRM) analysis were performed in a Rotor-Gene 6000 real-time 5-Plex HRM PCR thermocycler (Corbett Research Pty Ltd., Sydney, Australia) using the Rotor-Gene Q software (version 2.0.2) (Qiagen, Germantown, MD, USA). Cycling was performed with an initial denaturation step at 94 °C for 3 min followed by 45 cycles at 94 °C for 30 s, 55 °C for 35 s and 72 °C for 40 sec. An additional elongation step at 72 °C for 1 min was applied. The HRM was performed with an initial pre-melt conditioning of the PCR products at the first appropriate temperature for 90 sec followed by a melting ramp from 65 to 95 °C, with 0.2 °C increments every 2 s. The normalized raw and negative derivative of fluorescence (F) over temperature (T) (dF/dt) melting curves were used for sample comparisons. Detection sensitivity and reproducibility tests were confirmed by replicated DNA samples.

### 2.5. Data Analysis

The DNA fragment profiles for ISSR, RAPD and SCAR markers across samples were scored manually using binary data denoting presence (1) or absence (0). Similarly, the differences between male and female samples for the transcriptome-based markers were investigated based on a coefficient of variation (CV) of the HRM profiles with similarity > 70% scored as (1) when compared against the representative male and female reference curves and (0) denoting the absence of similarity. The raw data for all the analyses are presented in Table S1. Data were analyzed using GenAlEx 6.5 [37]. Band frequencies were calculated for each group (male and female). Genetic distance matrices were calculated, and further genetic analyses were performed, including information on number of different alleles (Na), number of effective alleles (Ne), Shannon’s Information Index (I), expected Heterozygosity (He), unbiased diversity (uh), gene diversity (GD), Analysis of Molecular Variance (AMOVA) and Principal Coordinates Analysis (PCoA). The genetic distance matrix was used for the cluster analysis based on the unweighted pair-group method with arithmetic averaging (UPGMA) [38] using MEGA X software version 4 [39]. Additionally, based on the scoring data, the polymorphism information content (PIC) for each marker was calculated using the online tool GDdom [40]. The STRUCTURE v2.3.4 package software [41] was applied to infer population structure based on the number of genetically homogeneous groups (K) in *P. lentiscus* var. *chia*. Three runs were performed with 10 repetitions with a burn-in period of 50,000 iterations followed by 100,000 Markov chain Monte Carlo (MCMC) iterations. Other parameters were set to the default values. The results of the analysis were uploaded to the Structure Harvester web page (Structure Harvester) to determine the appropriate K value using the ΔK ad hoc statistic [42].

## 3. Results

### 3.1. Assessment of the Sex-Linked ISSR, RAPD and SCAR Markers in Mastic Tree

To investigate the efficacy of 17 literature-based dominant markers for distinguishing the sex type between 49 male and 20 female mastic tree individuals, the overall PIC values were calculated and ranged from 0.25 to 0.43, with the most informative markers being the UBC841 (0.43) and (AC)_8_TA (Table S2). For the female individuals, the most informative markers were the PVF1-PVF2 (0.40) and UBC856 (0.40), whereas the least informative were the UBC860 (0.18) and UBC850 (0.11) (Table S3). Regarding the male individuals, the higher PIC values were observed in UBC841 (0.42) and (AC)_8_TA (0.36) and the least informative markers were the PVF1-PVF2 (0.20) and OPO08 (0.24).

The most effective separation of male and female individuals was achieved when the following ten primers (AC)_8_TA, (AC)_8_CG, SCO-08, UBC834, UBC827, UBC841, UBC811, UBC880, UBC856, UBC842 were used. A total of 169 loci were scored, of which 94.67% (160 bands) were polymorphic (P) for male and 82.84% (152 bands) for female individuals. The male individuals showed 16 private alleles against the female individuals which showed only 8 private alleles (Table 2). Overall, the genetic diversity of the total sample was relatively high (P = 88.8%, I = 0.452 ± 0.012, Nei’s genetic distance = 0.201). The male individuals showed higher Shannon’s Information Index (I) (0.510 ± 0.015) and diversity (h) (0.344 ± 0.011) compared to the females (0.393 ± 0.019 and 0.257 ± 0.014, respectively) (Table 2), with a mean diversity (h) for all loci of 0.300 ± 0.009.

**Figure 1:**
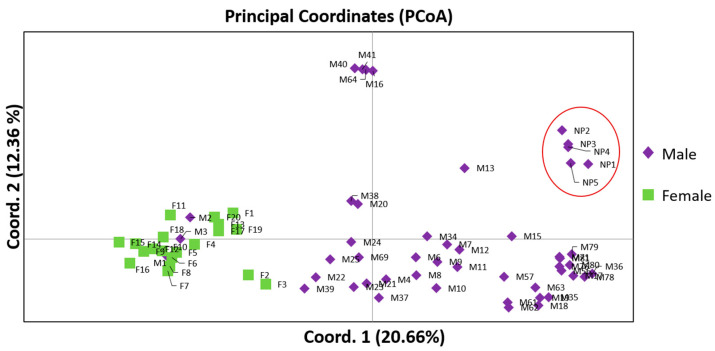
Principal Coordinate Analysis (PCoA) of 49 male and 20 female *P. lentiscus* var. *chia* individuals based on 169 polymorphic ISSR, RAPD and SCAR loci. The first two coordinates accounted for 33.03% of the total variance (axis 1 = 20.66%; axis 2 = 12.36%). The PCoA analysis demonstrate the separation of the two sex types (male vs. female) as distinct groups and differentiates the non-productive males in a sub-cluster. The red circle depicts the cluster of non-productive (NP) males.

Based on the Analysis of Molecular Variance (AMOVA), highly significant (*p* < 0.001) genetic differences were observed between the male and female mastic tree individuals (Table 3). The genetic differentiation between the sex types was statistically significant (Φ_ST_ = 0.263; *p* < 0.001), indicating that the two sex types could be effectively separated. The results indicated that 26% of the observed genetic diversity was attributed to among-population variability whereas 74% occurred within the genotypes (Table 3). The Principal Coordinates Analysis (PCoA) generated two major clusters, in which male and female individuals were clearly separated (Figure 1). Additionally, a sub-cluster of non-productive males was also depicted (Figure 1). The first two principal coordinates accounted for 33.04% of the total variation (Figure 1) and 43.7% of total variation accumulated when including the third coordinate. Among the different sex types, the female individuals showed reduced intrapopulation variability given the observed homogeneity (Figure 1, Table 2). The male individuals demonstrated high within population variability, whereas the individuals M1, M2 and M3 were grouped together with the females.

**Figure 2:**
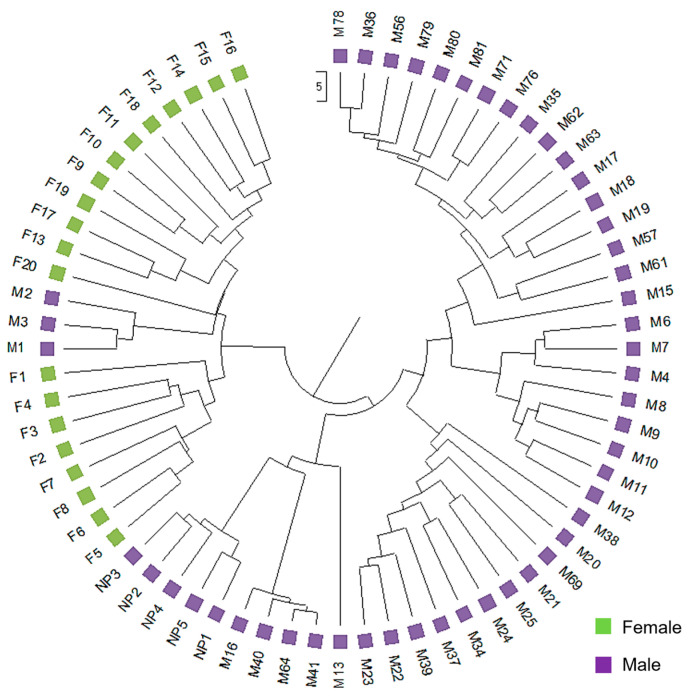
Genetic relationships of male and female *P. lentiscus* var. *chia* individuals based on the ISSR, RAPD and SCAR polymorphism analysis. The unweighted pairgroup method using arithmetic average (UPGMA) dendrogram illustrates the genetic relationships among 69 individuals of *P. lentiscus* var. *chia* analyzed with 169 loci obtained with ISSR, RAPD and SCAR markers.

**Figure 3:**
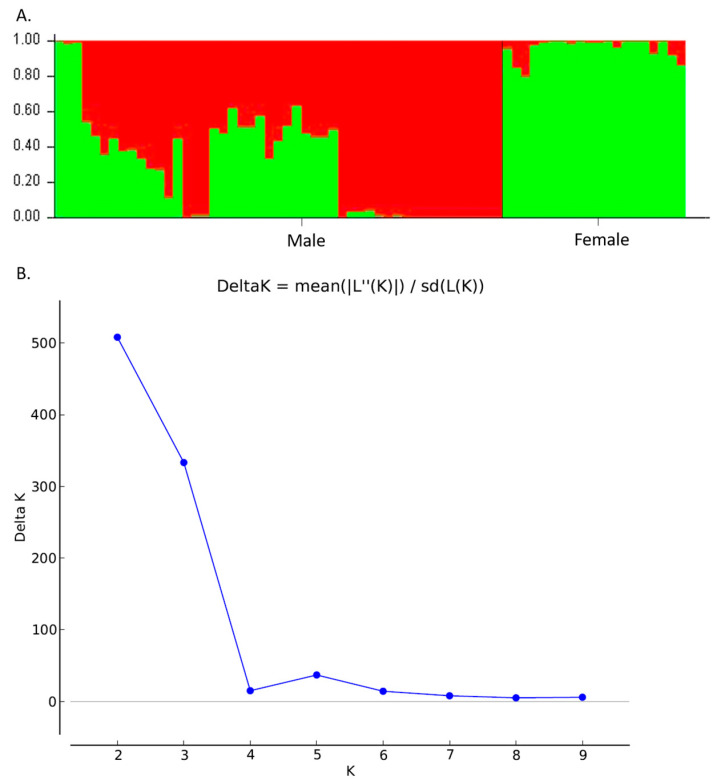
Genetic structure of 49 male and 20 female individuals of *P. lentiscus* var. *chia* by Bayesian inference. (**A**) Bar plot based on estimated membership coefficient values (Q) for maximum K value (Delta K = 2). Each bar represents a different individual and different colors represent the estimated membership coefficients. (**B**) Delta K (mean). Estimation of the number of clusters for K ranging from 1 to 10 by calculating delta K values.

The UPGMA dendrogram was consistent with the PCoA analysis illustrating that the mastic tree individuals of the same sex type were closely clustered (Figure 2). The dendrogram grouped the male and female individuals into two main clusters (Figure 2). The male cluster was split in two sister clades with the non-productive males forming a separate sub-cluster. The most probable number of genetic groups formed by the resulting data set, estimated using the Evanno method, was K = 2 [Lnprob (K) = −5449; Ln’(K) = 1069.6; Ln’’(K) = 442.93; and Delta K = 508.08) (Figure 3), indicating that the male and female individuals are separated, which is consistent with the PCoA (Figure 1) and UPGMA analyses (Figure 2). In all three analyses, the M1, M2 and M3 individuals were grouped with the female ones, whilst the other male individuals exhibited some gene exchange between the germplasms.

### 3.2. Genetic Diversity of Female Individuals and Different Cultivars of P. lentiscus var. chia

To investigate whether any potential associations exist between the sex identity and the genetic diversity of female individuals and different cultivars of *P. lentiscus* var. *chia* that are commonly assigned to distinct phenotypes based on specific morphological parameters, we used twelve ISSR markers, out of which seven were found to be polymorphic. The five cultivars were *Mavroschinos* (MV), *Votomos* (V), Maroulitis (ML), *Platiphyllos* (PL) and five plants that were not assigned to any phenotype and were characterized as non-productive (NP). Initially, for the ISSR analysis, all 20 female individuals and 49 males assigned to the different cultivars were assessed. More specifically, 134 loci were scored with 72.26% mean polymorphic loci at population level, ranging from 25.37% in the NP individuals and 64.18% in the PL to 88.06% in the ML populations (Table 4) and 83.58% in the female (F) population.

Diversity (h) was higher in the MV (0.323 ± 0.016), V (0.317 ± 0.014) and ML (0.349 ± 0.014) populations, with the ML being the most diverse, compared to PL and non-productive (NP) populations (Table 4), whereas the female population showed a moderate diversity (0.267 ± 0.015). Shannon’s Index ranged from 0.157 ± 0.024 to 0.511 ± 0.019 in populations NP and ML, respectively (mean I = 0.392 ± 0.011) (Table 4), with the Shannon diversity index in the female population being 0.406 ± 0.021. However, it was observed that, when included in the analysis, only the female population produced five unique bands; yet, when it was removed, the number of unique bands changed to three in the MV and one in the ML cultivars (Table 4).

The AMOVA of the ISSR markers when the female population was included in the analysis indicated significant differences among the populations at 21% and 79% within populations, with Φ_ST_ = 0.214 (*p* < 0.001) (Table S3). Based on these results and the Principal Coordinate Analysis (PCoA) and UPGMA analyses (Figure S1A,B), we observed that the genetic diversity among the cultivars was masked from the sex-related differences. The UPGMA analysis revealed closer association of the female individuals to most of the males of MV cultivar along with two males of the V and ML cultivars (Figure S1B). Cultivar PL and the NP males were both differentiated from the female population (Figure S1A,B).

**Figure 4:**
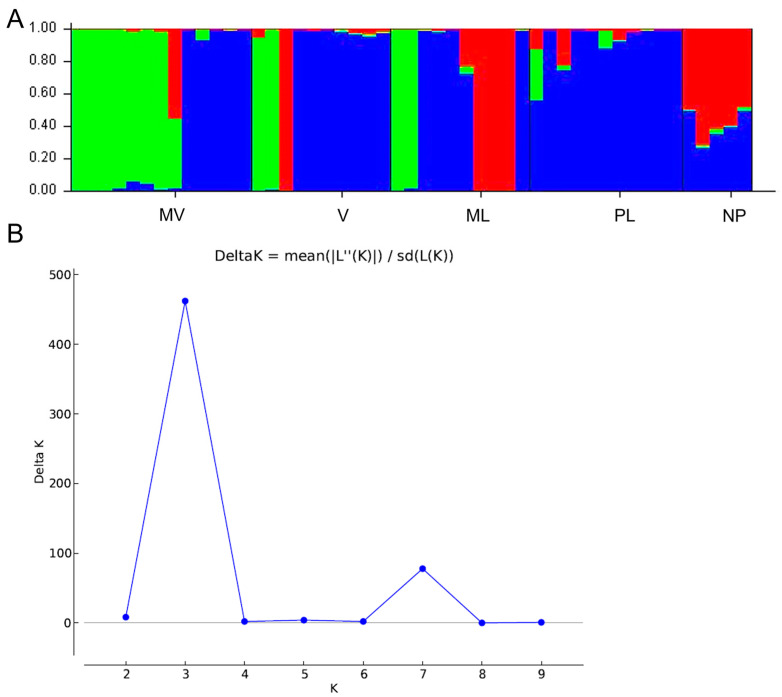
Genetic structure by Bayesian inference of 49 male individuals of *P. lentiscus* var. *chia* assigned to the following 5 cultivars: *Mavroschinos* (MV), *Votomos* (V), *Maroulitis* (ML), *Platiphyllos* (PL) and non-productive males (NP). (**A**) Bar plot based on estimated membership coefficient values (Q) for maximum K value (Delta K = 3). Each bar represents a different individual and different colors represent the estimated membership coefficients. (**B**) Delta K (mean). Estimation of the number of clusters for K ranging from 1 to 10 by calculating delta K values.

Therefore, we proceeded with further investigation of the genetic differences of the five male cultivars. The AMOVA analysis, indicated significant genetic differences (*p* < 0.001) among the five *P. lentiscus* var. *chia* populations (Table 5). The among-population differences were 14% of the total genetic diversity, supporting that the populations are relatively differentiated (Φ_ST_ = 0.144) and Shannon’s I, indicating genetic differentiation among populations (Table 5). Additionally, the genetic structure of 49 male individuals of *P. lentiscus* var. *chia* revealed that the most probable number of genetic groups formed by the resulting data set was K = 3 [Lnprob (K) = −2442.9; Ln’(K) = 497; Ln’’(K) = 359; and Delta K = 462) (Figure 4), indicating that the populations form three clusters. Structure analysis demonstrated that possibly V and ML have close relationships indicating that these genotypes may be a result of seed propagation. Some individuals assigned to the V population may belong to MV and ML and vice versa. Also, a moderate level of admixture was observed in the NP population, indicating to be the result of seed propagation derived from ML and V or PL (Figure 4).

**Figure 5:**
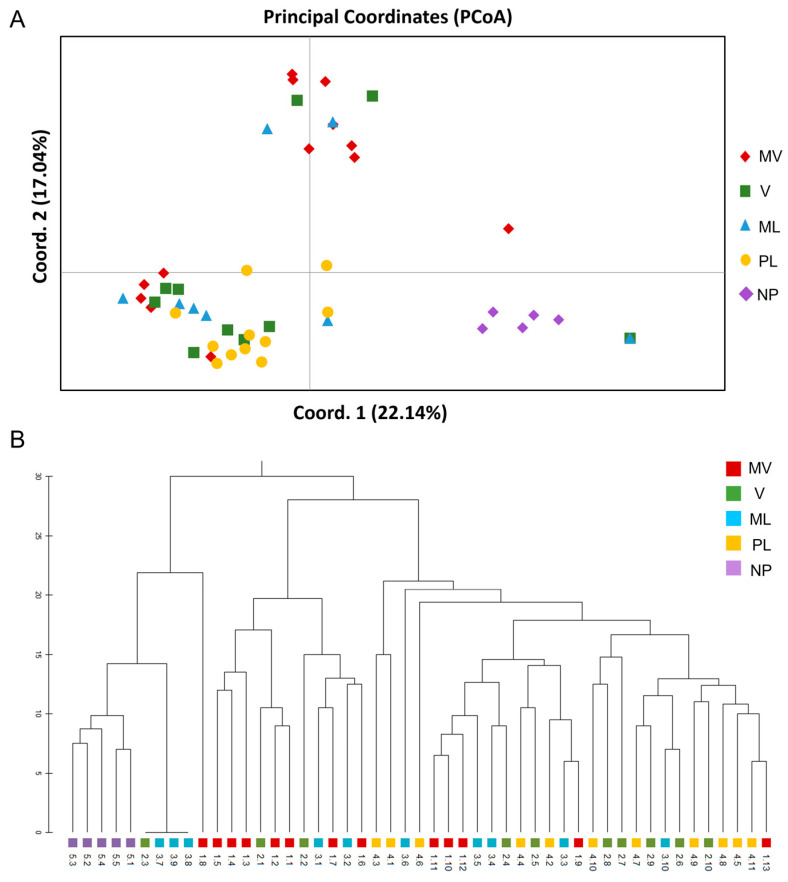
Genetic relationships of the 49 male individuals of five *P. lentiscus* var. *chia* cultivars, namely *Mavroschinos* (MV), *Votomos* (V), *Maroulitis* (ML), *Platiphyllos* (PL) and the non-productive males (NP) based on 134 polymorphic ISSR loci. (**A**) Principal Coordinate Analysis (PCoA). The first two coordinates accounted for 33.03% of the total variance (axis 1 = 22.14%; axis 2 = 17.04%). (**B**) The unweighted pairgroup method using arithmetic average (UPGMA) dendrogram based on the genetic distance matrix.

Furthermore, the PCoA results for the male cultivars (Figure 5A) depicted three major clusters, which were consistent with the UPGMA dendrogram (Figure 5B). The first two principal coordinates accounted for 33.03% of the total variance (axis 1 = 22.14%; axis 2 = 17.04%) (Figure 5A). Among the populations, the PL and NP were the most homogeneous, whereas the remaining populations demonstrated interaction among MV, V and ML (Figure 5A). These results were also supported by the Unweighted Pair Group Method with Arithmetic mean (UPGMA) clusters analysis with the five populations being grouped into at least three major clades (Figure 5B). The UPGMA method revealed the early divergence of clade A which included the NP population and some individuals of the ML and V populations showing less similarity to the other four populations. The latter clusters consisted of the MV, and some individuals of V and ML and the third clade was formed mainly by the PL and V populations with individuals of ML and MV populations which overlapped on the PCoA (Figure 5A).

### 3.3. Sex-Linked Marker Identification in Mastic Tree

To further examine whether it is possible to develop effective and sex-linked specific for the mastic tree genetic markers, the transcriptomes of six male (M), six female (F) and three monoecious (B) mature individuals of *P. lentiscus* var. *chia* were used. The novel markers were designed based on their homology to known important for flowering and sex differentiation genes, which demonstrated differential expression (DEGs) between different sexes or included unique per sex SNPs or EST-SSRs. More specifically, 543 genes that are important for flowering and sex determination were used to identify homologous loci within the 7189 transcripts with differences in SNPs, EST-SSRs or level of expression between the different sex groups. Six genes were identified with differences either in the sequence or expression level between the male and female mastic trees (Table 6) and 14 primers were designed (Table S4). Each marker demonstrated sex specific sequence differences (Figure S2), apart from ASC1, a marker derived by the differentially expressed homolog of the acetyl-coA synthetase gene, which was significantly downregulated in all productive male trees.

### 3.4. Assessment of the EST-SSR and DEG-Based Markers with HRM Analysis

The 14 primers (Table S4) developed for sex identification in *P. lentiscus* var. *chia* were used for high resolution melting (HRM) analysis by real-time PCR. Different HRM profiles of the eight markers, with male and female individuals as references, were used for scoring the derived binary data. Among the tested transcriptome-based markers, the eight most informative markers based on the polymorphism information content (PIC) values (Table S5 and Table 5) comprised six EST-SSRs, namely, the MYB3, SPL92, ELF61, ELF63, ELF66, Rpr21 and two DEG-based markers, the ACS1 and ACS2. In the initial analysis we included 34 male (M) and 5 non-productive male (NP) individuals along with 4 monoecious (B) and 20 female (F) *P. lentiscus* var. *chia* individuals. The 16 loci showed 85.42% polymorphism rate with a mean diversity (h) for all loci of 0.324 ± 0.026 and Shannon’s Index (I) 0.477 ± 0.035. The male population showed the highest polymorphism (100%) followed by the F (93.75%) and B (62.50%) populations, with no unique bands detected. The genetic structure analysis indicated that the individuals are clustered in two groups (the maximum Delta K = 2), which was corroborated by the results of genetic analysis with the PCoA showing partial separation of the two main sex types (male vs. female) as distinct groups with the first two coordinates accounting for 42.02% of the total variance (axis 1 = 29.24%; axis 2 = 12.67%). Most of the male NP individuals were grouped with the F population, whereas three out of four monoecious individuals were grouped with the male population (Figure S3). The AMOVA indicated relatively significant genetic differences (Φ_ST_ = 0.129, *p* < 0.022) between male, female, and monoecious populations, with 13% of the total genetic diversity being attributed to differences among populations.

Therefore, since the NP and B populations did not differentiate from the male and female populations, the NP and B individuals were removed from the analysis in order to investigate further the sex-linked associations. The eight transcriptome-based markers, developed herein, were able to better differentiate the male and female mastic trees in all 54 analyzed individuals (34 male and 20 female individuals). The (PIC) values ranged from 0.47 for the SPL92 marker to 0.32 for ELF63 marker with an average PIC value of 0.4 (Table S4), which indicated that the selected DEG-based markers were highly polymorphic. Regarding heterozygosity (h), for the female population, the highest (0.475 ± 0.02) value was observed for SPL92 and the lowest value (0.235 ± 0.14) for ELF63, whereas for the male population, the lowest value (0.278 ± 0.221) was observed for ACS1 and the highest for MYB3 (0.454 ± 0.039) (Table 7). The maximum Shannon’s Index (I) value (0.668 ± 0.0204) was observed for SPL92 marker, whilst the lowest was in Rpr21 (0.336 ± 0.336), both detected in the female population (Table 7).

**Figure 6:**
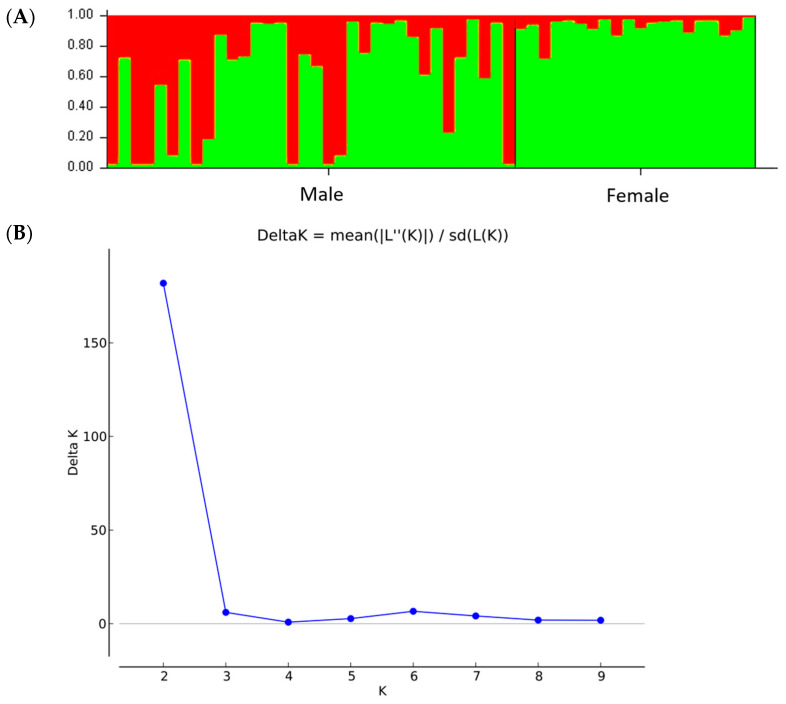
Genetic structure of 34 male and 20 female individuals of *P. lentiscus* var. *chia* by Bayesian inference. (**A**) Bar plot based on estimated membership coefficient values (Q) for maximum K value (Delta K = 2). Each bar represents a different individual and different colors represent the estimated membership coefficients. (**B**) Delta K (mean). Estimation of the number of clusters for K ranging from 1 to 10 by calculating delta K values.

**Figure 7:**
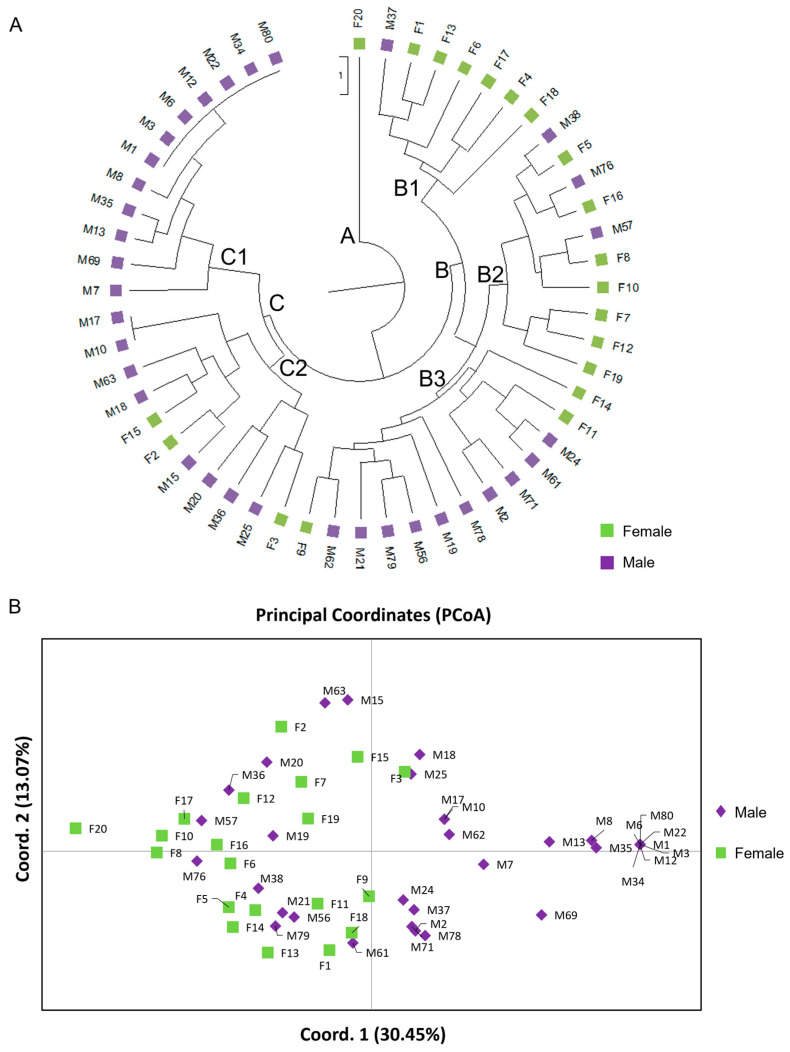
Genetic relationships of the 34 male and 20 female *P. lentiscus* var. *chia* individuals based on the developed EST-SSR and DEG-based polymorphism analysis. (**A**) The unweighted pair-group method using arithmetic average (UPGMA) dendrogram analyzed with the 18 loci. (**B**) Principal Coordinate Analysis (PCoA) for 14 polymorphic EST-SSR and 4 DEG-based loci. The first two coordinates accounted for 43.52% of the total variance (axis 1 = 30.45%; axis 2 = 13.07%). The PCoA analysis shows the partial separation of the two sex types (male vs. female) as distinct groups.

The genetic diversity of the total sample was high (P = 97%, I = 0.523 ± 0.033, Nei’s genetic distance = 0.153). Overall, 16 loci showed 96.88% polymorphism rate with a mean diversity (h) for all loci of 0.352 ± 0.026 and Shannon’s Index (I) 0.523 ± 0.033. There was one private allele found only in male population (Table 8). Results showed that the genetic diversity of male individuals was similar to that of the female population. The AMOVA showed highly significant (Φ_ST_ = 0.175, *p* < 0.001) genetic differences between male and female populations, with 18% of the total genetic diversity being attributed to differences among populations (Table 9). Structure analysis identified the maximum Delta K value being at K = 2 [Lnprob (K) = −802.1; Ln’(K) = 147.267; Ln’’(K) = 137.7; and Delta K = 1377) (Figure 6A,B), which was supported by the PCoA and UPGMA analysis (Figure 7). Interestingly, low levels of admixture were observed in the female population, whilst male populations demonstrated higher levels of admixture (Figure 6A).

The UPGMA analysis demonstrated the relatedness between male and female individuals, which were grouped mainly into two major clades (Figure 7A). Early divergence of female F20 (clade A) was observed along with the simultaneous emergence of clades B and C. The individuals in clade B were split into three sub-clades of which the sister clades B1 and B2 were mostly consisted of female and three male individuals (M37, M57, M76), and the third sub-clade B3 included mainly male individuals and three females (F9, F11, F14) (Figure 7A). The individuals in the B1 sub-clade were clustered together in the center of the lower right quartile of the PCoA, whereas the closely related B2 and B3 clades overlapped on the left quartiles of the PCoA (Figure 7B). Clade C was composed of male individuals and three females (F2, F3, F15), with individuals in the C1 being distinctively clustered in the extreme right quartile and the C2 in the center of the right quartile of the PCoA, respectively (Figure 7A). Similarly, relative discrimination between male and female individuals was observed on a two-dimensional multivariate space, which were separated by the central axis of coordinate 1 explaining the 30.45% of the total variance among the 54 *P. lentiscus* var. *chia* individuals (Figure 7B). The first two coordinates of the PCoA explained 43.52% of the total variance (Figure 7B), whilst the first three coordinates explained up to 55.42% of the cumulative variance.

## 4. Discussion

*P. lentiscus* var. *chia* is of high economic importance for the pharmaceutical properties of the mastic gum, which is produced by the male trees in the island of Chios. However, the male mastic trees can reach full sexual maturity after approximately six years, when mastic production begins. Thus, distinguishing the male trees in early growth stages is important aim for the mastic tree breeding attempts, considering that genetically assisted breeding for productivity prerequisites sex identification of trees prior to the development of productivity associated genetic markers. Sex identification in Pistacia species has mainly focused on *P. vera* L. and other related species, such as *P. eurycarpa* and *P. atlantica* [6,7,9,14,17,20,21,43] using various sex-linked DNA markers. Despite the importance of *P. lentiscus* var. *chia*, the genome of this species is still largely unknown, and thus far the use of *P. vera* as reference genome is available. Limited studies have also been performed mainly for genotyping in *P. lentiscus* genotypes with the use of ISSR, RAPD and SSR markers [26] and *P. lentiscus* var. *chia* using ISSR and RAPD markers [27,28]. To our knowledge, this is the first report regarding: (i) transcriptome-based marker coupled with HRM analysis specifically developed for this species and especially for the *chia* variety, using male and female populations along with identified monoecious individuals, to demonstrate a cost-effective method for sex identification that can be applied with minimum infrastructure and (ii) effective sex differentiation in *P. lentiscus* var. *chia* with 100% accuracy using a combination of different sex-linked ISSR, RAPD and SCAR markers. Additionally, investigating whether sex identity is associated with genetic variations and population structure we were able to examine the genetic relations amongst phenotypic cultivars of *P. lentiscus* var. *chia*.

Our results indicated 100% accuracy on sex differentiation in *P. lentiscus* var. *chia* genotypes based on a combination of one SCAR and nine ISSR markers. These markers have been previously used either in *P. vera*, such as the SCO-08 [23], (AC)_8_CG and (AC)_8_TA [44], or in *P. lentiscus* var. *chia*, such as the UBC811, UBC827, UBC834 UBC841, UBC842, and UBC856 [27,28] along with the UBC880, which we also included in this study. Our results are in accordance with the effective sex separation on *P. lentiscus* genotypes based on ISSR markers [26]. The 169 loci for a Φ_ST_ at 0.263 were more than adequate for estimating population structure [45]. The population structure analysis was in accordance with the PCoA and the UPGMA dendrogram demonstrated a clear separation of male and female genotypes. Interestingly, the non-productive males formed a distinct group, closer related to the male individuals. The male individuals showed higher genetic diversity compared to the females, which exhibited reduced intrapopulation variability. Comparable results were also demonstrated in genetic diversity studies in different male cultivars and female *P. lentiscus* var. *chia* genotypes based on ISSR markers [27,28].

Sex-related differences between male and female individuals masked the genetic diversity among 20 females and the 49 males previously assigned to the 5 phenotypic cultivars, *Mavroschinos* (MV), *Votomos* (V), *Maroulitis* (ML), and *Platiphyllos* (PL). This effect has also been observed in *P. vera*, 20 male and 20 female genotypes where the genetic distance was found to be mainly affected by the sex type rather than the intrapopulation distances [24]. Herein, the female individuals showed closer genetic association to most of the males assigned to *Mavroschinos* along with two males of the *Votomos* and *Maroulitis* cultivars, whereas the males of *Platiplyllos* were further distant. This can be attributed to the higher diversity demonstrated by the *Mavroschinos*, *Maroulitis* and *Votomos* cultivars compared to *Platiplyllos*. Additionally, the non-productive (NP) individuals demonstrated low genetic diversity and were again clustered separately from the male population and not assigned to any phenotypic cultivar. Comparatively to our results, studies on genetic diversity of the same morphological cultivars confirm the existence of genetic heterogeneity [27,28], which is possibly the effect of either vegetative propagation or intercrossing indicating hybridization.

Based on genetic structure analysis, three main genetic groups were formed, (i) *Mavroschinos*, which seems to share genetic material with males of the *Votomos* (M2 and M8) and *Maroulitis* (M9 and M10) cultivars, possibly a result of mis-assigned individuals to these cultivars, (ii) *Platiphyllos*, which was the most homogeneous cultivar and (iii) *Maroulitis* with a cluster including M40, M41, M64 and M16, which has been possibly mis-assigned as *Votomos*. Furthermore, *Votomos* and *Platiphyllos* seem to interact and be genetically related, which could possibly indicate that these genotypes may have derived from vegetative propagation or may have a common genetic background. The non-productive population was also more homogeneous and presented a moderate level of admixture indicating a degree of intercrossing between *Maroulitis* and *Platiphyllos* and possibly *Mavroschinos*. Interestingly, *Mavroschinos* and *Maroulitis* presented three and one unique bands, respectively. The unique specific bands amplified in the two cultivars could potentially help in certification of these cultivars, yet further research, in a large germplasm collection, is required for validation of reliable markers.

Sex-linked marker identification has been mainly reported in *P. vera*, mainly given the existence of molecular tools and the high-quality genome assembly [14], yet not with 100% accuracy in other related species [6,25]. Research on marker development for sex identification in Pistacia genus has mainly focused on simple sequence repeat (SSR) in *P. vera* and their transferability in other Pistacia species [46], expressed sequence tag-derived (EST)-SSR in *P. chinensis* [25] and single nucleotide polymorphism in *P. vera* (SNP) [7]. Herein, we report the first EST-SSR and DEG-based markers’ design on male, female and monoecious *P. lentiscus* var. *chia* genotypes and their application using RT-PCR coupled with high-resolution melting (HRM) analysis for a cost-effective approach. Our results showed that the genetic diversity of male and female populations was relatively high and that male populations demonstrated higher levels of admixture. Interestingly, most of the non-productive male individuals were grouped with the females, whereas monoecious individuals were grouped with the males. Monoecious trees possibly originated from female trees [15], given that in the ZW/ZZ sex-determination system the females are the heterozygotes [14]. Studies on monoecious occurrence have shown that gender types (male, female or monoecious) in *P. chinensis* Bunge were unstable in successive years [16], and this could potentially be attributed to sexual plasticity as a response to environmental stresses and sex determination mechanism [15].

The designed novel primers did not overlap with any of the primers reported in literature [10,15,20,47]. The eight most informative markers comprised six EST-SSRs and two DEG-based markers. More specifically, the six EST-SSRs based novel markers developed in the present study, were all found on DNA binding proteins, that are known for their relation to sex determination and flowering pathways in plants. Several MYB family members have been shown to play a role in the regulation of several cellular processes, as well as in responses to biotic and abiotic stress [48], and in sex determination [30]. Interestingly, the association of MYB with the SWI/SNF ATP-dependent chromatin remodeling complex could also indicate a possible temperature dependency of the sex determination in mastic trees [49]. The *SUF4* is a suppressor of *FRI4* and a transcription factor (TF) required for delayed flowering in winter-annual Arabidopsis and has been characterized as a differentiation and developmental protein, as well as a DNA-binding TF with an activity in histone H3-K4 methylation [50]. The *SPL9* are Squamosa Promoter Binding-Like Proteins and SBP TF, that have been connected to early flowering and other developmental functions [51]. The *ELF6* is an Early Flowering (6) TF of the jumonji (jmj) protein family, which is involved in histone demethylation, resetting the embryo epigenetic memory [50]. Lastly, the *Rpr2* is a DOF-zinc finger DNA-binding domain with a TF function involved in flowering regulation and responses to biotic and abiotic stresses [52].

The two DEG-based markers that showed significant polymorphism information content (PIC) values, ACS1 and ACS2, were both found on a homologous to the acetyl-CoA synthetase (*ACS*) gene, a gene that was found to be significantly downregulated in productive male individuals. ACS is peculiar in a sense that it is not a DNA binding protein, and it is mostly related to metabolism and not to sex differentiation. ACS in plants has been shown to be a component of a two-enzyme system that plants use to maintain acetate homeostasis [53]. Our findings regarding ACS could indicate an acetate homeostasis dependency of sex determination that is visible on a transcription level. Interestingly, acetyl-coA has been found to play a significant role in many regulatory processes associated with acetylation of controlling components (such as histone acetylation or N-terminal and/or amino acid side-chain acetylation). Hence, the post-translational acetylation mechanism may enable plants to respond to shifting environmental and developmental conditions, which could affect the sex determination whilst controlling the acetyl-CoA and acetate balance [53]. Regarding the lack of visible sequence differences between the sexes for ACS1 on a transcript level it might indicate a differential mRNA maturation, that could affect the half-life of the mRNA and thus, its detection and translation.

The percentage of true positives for sex identification was more than 80% for males and 70% for females. It is noteworthy that sex-linked SNP loci markers were unable to successfully identify the sex in wild *Pistacia* species, including *P. lentiscus* [6,7]. Additionally, the transferability of sex-linked markers identified in *P. vera* was found to be the lowest in *P. lentiscus* L. [46], which could be due to the larger genetic distance between the two species [11,54]. This could be an effect of the genetic or epigenetic control of sex regulation that may alter the related gene(s) [55]. Therefore, the novel nature and the ability of the transcriptome-based markers to accurately assign the true positive male and female trees could provide insights in the sex determination mechanisms of *P. lentiscus*.

## 5. Conclusions

Marker-assisted selection (MAS) of male mastic tree individuals could be an important tool for future mastic tree breeding programs. The novel species-specific polymorphic EST-SSR and SNP markers developed in this study will be useful for further genetic studies and breeding efforts of *P. lentiscus* var. *chia* genetic resources. To our knowledge, this is the first report regarding the development of transcriptome-based EST-SSRs and SNPs specific for the *P. lentiscus* var. *chia*, using male, female, and monoecious individuals, to demonstrate a cost-effective method for sex identification that can be applied with minimum infrastructure using the PCR-HRM technique. The designed novel primers were based on the differential expression between the different sex types or included unique per sex-type SNPs or EST-SSRs. The eight most informative markers comprised six EST-SSRs that were found on DNA binding proteins associated with sex determination and flowering pathways, whereas the two DEG-based markers were both found on a homologous to the acetyl-CoA synthetase gene, that was found to be significantly downregulated in productive male individuals which may enable plants to respond to shifting environmental and developmental conditions. The novel nature and the ability of the transcriptome-based markers to accurately detect the true positive males with 80% accuracy could provide insights in the sex determination mechanisms of *P. lentiscus*.

Moreover, using a combination of different sex-linked ISSR and SCAR markers we were able to identify the male from female individuals of *P. lentiscus* var. *chia* with 100% accuracy. The sex-related differences between male and female individuals masked the genetic diversity among the male phenotypic cultivars, *Mavroschinos*, *Votomos*, *Maroulitis*, and *Platiphyllos*. When only the male individuals were examined, we identified three potential groups (i) *Mavroschinos*, (ii) *Platiphyllos* and (iii) *Maroulitis*. This is also the first genetic diversity report on non-productive population that presented a moderate level of admixture indicating a degree of intercrossing between *Maroulitis* and *Platiphyllos* and possibly *Mavroschinos*. The unique specific bands amplified in *Mavroschinos* and *Maroulitis* cultivars could potentially assist in the certification processes of these cultivars, yet further research, in a larger germplasm collection, is required for validation of the tested markers. Information on genetic diversity of the unexplored *P. lentiscus* var. *chia* genetic resources is important for selecting high-yielding genotypes adapted to different environmental conditions and for increasing product potential in future breeding programs. Future research will focus on developing molecular markers for high productivity and on associating the sex-related differences with the yield and quality of mastic gum.

## Figures and Tables

**Table 1:** Literature-based molecular markers used for sex identification in male and female mastic trees with the respective sequences and the annealing temperature used in this study.

Primers	Type	Sequence	Species	Reference	Ta °C
OPO-08	RAPD	5′-CCTCCAGTGT-3′	*P. vera*	[20,23]	32
SCO-08 F	SCAR	5′-CCTCCAGTGTGAATCAAGTAAAC-3′	*P. vera*	[20]	56
SCO-08 R		5′-CCTCCAGTGTTATGTAATACCAAAA-3′			
PVF1 F	SCAR	5′-GTCGTAGATGAAAACACC-3′	*P. vera P. khinjuk, P. atlantica, P. vera*	[9,21]	Touchdown 60–50 and 51
PVF2 R		5′-TAATAGAAGCCATAGA-3′			
(AC)8CG	ISSR	5′-ACACACACACACACACCG-3′	*P. vera*	[22]	37
(AC)8TA	ISSR	5′-ACACACACACACACACTA-3′			37
UBC807	ISSR	5′-AGAGAGAGAGAGAGAGT-3	*P. lentiscus* var. *chia*	[28]	54
UBC811	ISSR	5′-GAGAGAGAGAGAGAGAC-3	*P. lentiscus* var. *chia*	[28]	50.5
UBC823	ISSR	5′-TCTCTCTCTCTCTCTCC-3	*-*	-	49.5
UBC827	ISSR	5′-ACACACACACACACACG-3	*P. lentiscus* var. *chia*	[28]	47.5
UBC834	ISSR	5′-AGAGAGAGAGAGAGAGYT-3	*P. lentiscus* var. *chia*	[28]	55
UBC841	ISSR	5′-GAGAGAGAGAGAGAGAYC-3	*P. lentiscus* var. *chia*	[28]	49.5
UBC842	ISSR	5′-GAGAGAGAGAGAGAGAYG-3′	*P. lentiscus* var. *chia*	[27]	56
UBC850	ISSR	5′-GTGTGTGTGTGTGTGTYC-3′	*P. lentiscus* var. *chia*	[27]	52
UBC856	ISSR	5′-ACACACACACACACACYA-3′	*P. lentiscus* var. *chia*	[27,28]	57.5
UBC860	ISSR	5′-TGTGTGTGTGTGTGTGRA-3	-	-	48.5
UBC880	ISSR	5′-GGAGAGGAGAGGAGA-3	-	-	51
UBC891	ISSR	5′-HVHTGTGTGTGTGTGTG-3′	-	-	52.5

**Table 2:** Genetic diversity parameters for male and female *P. lentiscus* var. *chia* individuals based on ISSR, RAPD and SCAR markers. Data are presented as mean values ± standard errors (SE) for each population (POP).

POP	N	NPB	Number Unique Bands	Na	Ne	I	h	P%
Male	49	160	16	1.893 ± 0.035	1.594 ± 0.024	0.510 ± 0.015	0.344 ± 0.011	94.67
Female	20	152	8	1.728 ± 0.049	1.426 ± 0.027	0.393 ± 0.019	0.257 ± 0.014	82.84
Average	34.5 ± 0.79	156	-	1.811 ± 0.030	1.510 ± 0.019	0.452 ± 0.009	0.300 ± 0.009	88.76 ± 5.92

N = numbers of individuals, NPB = numbers of polymorphic bands, Na = Number of different alleles, Ne = Number of effective alleles = 1/(p^2^ + q^2^), I = Shannon’s information index, h = Diversity = 1 − (p^2^ + q^2^), P% = percentage of polymorphic bands.

**Table 3:** AMOVA analysis of the male and female *P. lentiscus* var. *chia* populations (POP), based on ISSR, RAPD and SCAR markers.

Source	df	Sum Squares	Mean Square	Estimated Variance	%	Φ_ST_
Among POP	1	308.000	308.000	9.868	26%	0.263 *p* < 0.001
Within POP	67	1856.203	27.705	27.705	74%	
Total	68	2164.203	-	37.572	100%	

Df = Degrees of freedom.

**Table 4:** Genetic diversity of 5 populations of 49 *P. lentiscus* var. *chia* individuals. The five cultivars were the *Mavroschinos* (MV), *Votomos* (V), *Maroulitis* (ML), *Platiphyllos* (PL) and non-productive males (NP). Data are presented as mean values ± standard errors (SE) for each population (POP).

POP	N	NPB	No. Unique Bands	No. LComm Bands (≤50%)	Na	Ne	I	h	%P
MV	13	121	3	5	1.769 ± 0.053	1.574 ± 0.032	0.476 ± 0.021	0.323 ± 0.016	86.57
V	10	115	0	3	1.716 ± 0.060	1.541 ± 0.028	0.472 ± 0.019	0.317 ± 0.014	85.82
ML	10	118	1	1	1.761 ± 0.056	1.618 ± 0.029	0.511 ± 0.019	0.349 ± 0.014	88.06
PL	11	105	0	2	1.425 ± 0.071	1.391 ± 0.032	0.344 ± 0.024	0.23 ± 0.017	64.18
NP	5	47	0	1	0.604 ± 0.075	1.197 ± 0.031	0.157 ± 0.024	0.109 ± 0.016	25.37
Mean	9.8 ± 0.102	101.2	-	-	1.455 ± 0.033	1.464 ± 0.015	0.392 ± 0.011	0.266 ± 0.008	70 ± 12

N = numbers of individuals, NPB = numbers of polymorphic bands, No. LComm Bands (≤50%) = Number of Locally Common Bands (Freq. ≥ 5%) found in ≤50% populations, Na = Number of different alleles, Ne = Number of effective alleles = 1/(p^2^ + q^2^), I= Shannon’s information index, h = Diversity = 1 − (p^2^ + q^2^), P%= percentage of polymorphic bands.

**Table 5:** AMOVA analysis of 5 cultivars of 49 *P. lentiscus* var. *chia* individuals. The five cultivars—populations (POP) were the *Mavroschinos* (MV), *Votomos* (V), *Maroulitis* (ML), *Platiphyllos* (PL) and non-productive males (NP).

Source	df	SS	MS	Est. Var.	%	Φ_ST_
Among Pops	4	222.071	55.518	3.565	14%	0.144 *p* < 0.001
Within Pops	44	933.357	21.213	21.213	86%	
Total	48	1155.429	-	24.778	100%	

Df = Degrees of freedom.

**Table 6:** Suggested genetic markers and amplicon specifications for high resolution melting curve analysis, which demonstrated differential expression (DEGs) between the different sexes of *P. lentiscus* var. *chia* or include unique per sex SNPs or EST-SSRs.

Reference Gene	Name	Description	Pathway	Dataset	Pident	Evalue
AT1G21700	*MYB*	Specific subunit of BRM-associated SWI/SNF (BAS) complexes | Chromatin remodelling	-	SNP/EST-SSR	74.045	2.37 × 10^−126^
AT1G30970	*SUF4*	Suppressor of FRI 4 | transcription factor required for delayed flowering in winter-annual Arabidopsis	-	SNP/EST-SSR	81.361	2.48 × 10^−73^
AT3G57920	*SPL9*,*15*	Squamosa Promoter Binding Protein-Like 15 | SBP transcription factor	Vegetative & reproductive phase change	SNP/EST-SSR	92.5	9.44 × 10^−8^
AT5G04240	*ELF6*	Early Flowering 6 | transcription factor jumonji (jmj) family protein involved in histone demethylation	-	SNP/EST-SSR	81.602	0
AT5G66940	*Rpr*2	DOF-zinc finger DNA-binding domain transcription factor	-	EST-SSR	84.416	7.37 × 10^−37^
AT5G36880	*ACS*	Plastidic acetyl-coA synthetase | Prevention of toxic accumulation of fermentation products	Pyruvate dehydrogenase bypass pathway	DEG	99.725	0

**Table 7:** Diversity parameters for 6 EST-SSR and 2 DEG-based loci studied in 34 male and 20 female *P. lentiscus* var. *chia* individuals. Data are presented as mean value ± standard error (SE) for each locus.

Locus	POP	I	h	Ne	PIC
*MYB3*	Male	0.646 ± 0.0402	0.454 ± 0.039	1.841 ± 0.131	0.454 ± 0.028
	Female	0.587 ± 0.0243	0.397 ± 0.023	1.662 ± 0.0621	0.397 ± 0.016
*SPL92*	Male	0.557 ± 0.0487	0.371 ± 0.044	1.598 ± 0.112	0.371 ± 0.031
	Female	0.668 ± 0.0204	0.475 ± 0.02	1.907 ± 0.073	0.475 ± 0.014
*ELF61*	Male	0.579 ± 0.0704	0.398 ± 0.065	1.663 ± 0.177	0.392 ± 0.05
	Female	0.555 ± 0.133	0.375 ± 0.12	1.661 ± 0.319	0.375 ± 0.085
*ELF63*	Male	0.474 ± 0.175	0.309 ± 0.148	1.516 ± 0.324	0.309 ± 0.105
	Female	0.380 ± 0.182	0.235 ± 0.14	1.353 ± 0.247	0.235 ± 0.100
*ELF66*	Male	0.436 ± 0.213	0.284 ± 0.173	1.483 ± 0.358	0.284 ± 0.122
	Female	0.629 ± 0.0183	0.437 ± 0.018	1.779 ± 0.055	0.4375 ± 0.012
*Rpr21*	Male	0.524 ± 0.106	0.344 ± 0.093	1.557 ± 0.222	0.344 ± 0.066
	Female	0.336 ± 0.336	0.24 ± 0.24	1.461 ± 0.461	0.24 ± 0.170
*ACS1*	Male	0.412 ± 0.279	0.278 ± 0.221	1.527 ± 0.466	0.278 ± 0.156
	Female	0.531 ± 0.031	0.347 ± 0.027	1.535 ± 0.065	0.348 ± 0.019
*ACS2*	Male	0.488 ± 0.189	0.323 ± 0.162	1.566 ± 0.374	0.323 ± 0.114
	Female	0.558 ± 0.135	0.377 ± 0.123	1.671 ± 0.3289	0.435 ± 0.042

I = Shannon’s information index, h = Diversity = 1 − (p^2^ + q^2^), Ne= Number of effective alleles = 1/(p^2^ + q^2^), P% = percentage of polymorphic bands.

**Table 8:** Genetic diversity parameters for male and female *P. lentiscus* var. *chia* individuals. Data are presented as mean values ± standard errors (SE) for each population.

Population	N	NPB	Unique Bands	Na	Ne	I	h	P%
Male	34	16	1	2 ± 0	1.594 ± 0.081	0.514 ± 0.046	0.344 ± 0.037	100
Female	20	15	0	1.875 ± 0.125	1.629 ± 0.077	0.531 ± 0.048	0.361 ± 0.037	93.75
Mean ± SE	27 ± 1.257	15.5	-	1.938 ± 0.063	1.611 ± 0.055	0.523 ± 0.033	0.352 ± 0.026	96.88 ± 3.13

N = numbers of individuals, NPB = numbers of polymorphic bands, Na = Number of different alleles, Ne = Number of effective alleles = 1/(p^2^ + q^2^), I = Shannon’s information index, h = Diversity = 1 − (p^2^ + q^2^), P% = percentage of polymorphic bands.

**Table 9:** AMOVA analysis of the male and female *P. lentiscus* var. *chia* populations (POP) based on the EST-SSR and DEG-Based Markers with HRM Analysis.

Source	df	SS	MS	Est. Var.	%	Φ_ST_
Among Pops	1	18.468	18.468	0.618	18	0.175 *p* < 0.001
Within Pops	52	151.347	2.910	2.910	82	
Total	53	169.815	-	3.528	100	

Df = Degrees of freedom.

## Data Availability

The raw transcriptomics data that supported the development of the novel markers for sex identification in *P. lentiscus* var. *chia* have been released in BioProject with the code PRJNA918300 and the title “Transcriptome analysis of different sex and productivity groups in *Pistacia lentiscus* trees”. The record will be publicly available at the date 29 June 2024 or upon publication. Please refer to the reviewer link provided bellow to review the raw data: https://dataview.ncbi.nlm.nih.gov/object/PRJNA918300?reviewer=scgl0fdhi7h2dcrhu7419bc5ua.

## Supplementary Materials

The following supporting information can be downloaded at: https://www.mdpi.com/article/10.3390/genes15050632/s1, Figure S1: Genetic relationships of 20 female and 49 male individuals of five *P. lentiscus* var. *chia* cultivars based on the ISSR polymorphism analysis. A. Principal Coordinate Analysis (PCoA) based on 134 polymorphic ISSR loci. The first two coordinates accounted for 33.47% of the total variance (axis 1 = 18.26%; axis 2 = 15.21%). B. The unweighted pair group method using arithmetic average (UPGMA) dendrogram of the six populations.; Figure S2: Primer design for the eight most informative markers based on the polymorphism information content (PIC) values (Table S4 and Table 5) comprised six EST-SSRs and two DEG-based markers (MYB3, SPL92, ELF61, ELF63, ELF66, Rpr21, ACS1 and ACS2) found in mastic trees. Letters stand for Q = Query, M = Male, F = Female, B = Monoecious. The product is presented with bold letters, while the primers used are shown in red. Polymorphisms are highlighted in yellow, while deletions found in some of the repeats are highlighted in grey; Figure S3: Principal Coordinate Analysis (PCoA) of the 34 male and 5 non-productive male individuals along with 4 bisexual and 20 female *P. lentiscus* var. *chia* individuals based on the developed 14 EST-SSR and four DEG-based polymorphic loci. The first two coordinates accounted for 42.02% of the total variance (axis 1 = 29.24%; axis 2 = 12.67%). The PCoA analysis shows the partial separation of the two sex types (male vs. female) as distinct groups. Most of the male NP individuals are grouped with the F population, whereas most three out of four bisexual individuals are grouped with the male population. Table S1: Raw data of the sex-linked literature-based ISSR, RAPD and SCAR markers and the novel developed transcriptome-based markers; Table S2: Average PIC and standard error (SE) values for the ISSR, RAPD and SCAR markers used for distinguishing male and female mastic trees; Table S3: AMOVA analysis of 20 female individuals and the 49 males assigned to the different *P. lentiscus* var. *chia* cultivars; Table S4: Transcriptome-based primers designed based on the markers selected; Table S5: Average PIC and standard error (SE) values for the transcriptome-based markers used for distinguishing male and female mastic trees.
