# Supplementary material for: Identification of Sex-Associated Genetic Markers in Pistacia lentiscus var. chia for Early Male Detection
Source: Genes (Basel). 2024 May 16;15(5):632. doi: 10.3390/genes15050632 (PMC11120708; doi:10.3390/genes15050632)
Supplement: Supplementary file 1 [file genes-15-00632-s001.zip › genes-2987894-Supplemental material.pdf]

Article

Identification of Sex-Associated Genetic Markers in *Pistacia lentiscus* var. *chia* for Early Male Detection

Evangelia Stavridou [1,2], Ioanna Karamichali [2], Evangelos Siskas [1], Irini Bosmali [2], Maslin Osanthanunkul [3,4] and Panagiotis Madesis [2,5,*]

[1] Department of Botany, School of Biology, Aristotle University of Thessaloniki, 54124 Thessaloniki, Greece
[2] Laboratory of Agrobiotechnology and Molecular Plant Breeding, Institute of Applied Biosciences (INAB), Center for Research and Technology (CERTH), 57001 Thessaloniki, Greece
[3] Department of Biology, Faculty of Science, Chiang Mai University, Chiang Mai 50200, Thailand
[4] Research Centre in Bioresources for Agriculture, Industry and Medicine, Chiang Mai University, Chiang Mai 50200, Thailand
[5] Laboratory of Molecular Biology of Plants, Department of Agriculture Crop Production and Rural Environment, School of Agricultural Sciences, University of Thessaly, 38446 Volos, Greece
* Correspondence: pmadesis@uth.gr

Table S2: Average PIC and standard error (SE) values for the ISSR, RAPD and SCAR markers used for distinguishing male and female mastic trees.

Marker	Average PIC value Males	Average PIC value females	Average GD value overall
AC8CG	0.2661±0.0432	0.2701±0.0237	0.3407±0.0428
AC8TA	0.3656±0.0439	0.2262±0.0465	0.4192±0.0358
UBC860	0.3637±0.0221	0.1809±0.0399	0.3248±0.0246
UBC811	0.3659±0.0346	0.2996±0.0405	0.3643±0.0337
UBC891	0.3036±0.0341	0.1881±0.041	0.2872±0.0353
UBC823	0.3015±0.0302	0.2918±0.0494	0.3317±0.0351
UBC880	0.3851±0.0279	0.2511±0.0387	0.3692±0.0292
UBC807	0.2995±0.0399	0.2036±0.0405	0.3032±0.0391
UBC841	0.4244±0.0204	0.265±0.0389	0.4291±0.0155
UBC834	0.3128±0.0401	0.2921±0.034	0.3529±0.0277
UBC827	0.3181±0.0296	0.307±0.0338	0.3512±0.0225
UBC856	0.3304±0.0319	0.3948±0.0242	0.3715±0.0262
UBC850	0.2601±0.0461	0.1142±0.0237	0.2461±0.0478
UBC842	0.3397±0.0233	0.2716±0.0291	0.3652±0.0248
OPO08	0.2362±0.0342	0.3113±0.0459	0.2912±0.0361
SCO08	0.346±0.0672	0.2656±0.0608	0.4±0.0513
PVF1PVF2	0.1991±0.0471	0.3973±0.0359	0.3133±0.0438

Table S3: AMOVA analysis of 20 female individuals and the 49 males assigned to the different *P. lentiscus* var. *chia* cultivars.

Source	df	SS	MS	Est. Var.	%	Φ_{ST}
**Among Pops**	5	414.202	82.840	5.593	21%	0.214 *p*<0.001
**Within Pops**	63	1290.957	20.491	20.491	79%	
**Total**	68	1705.159		26.084	100%	

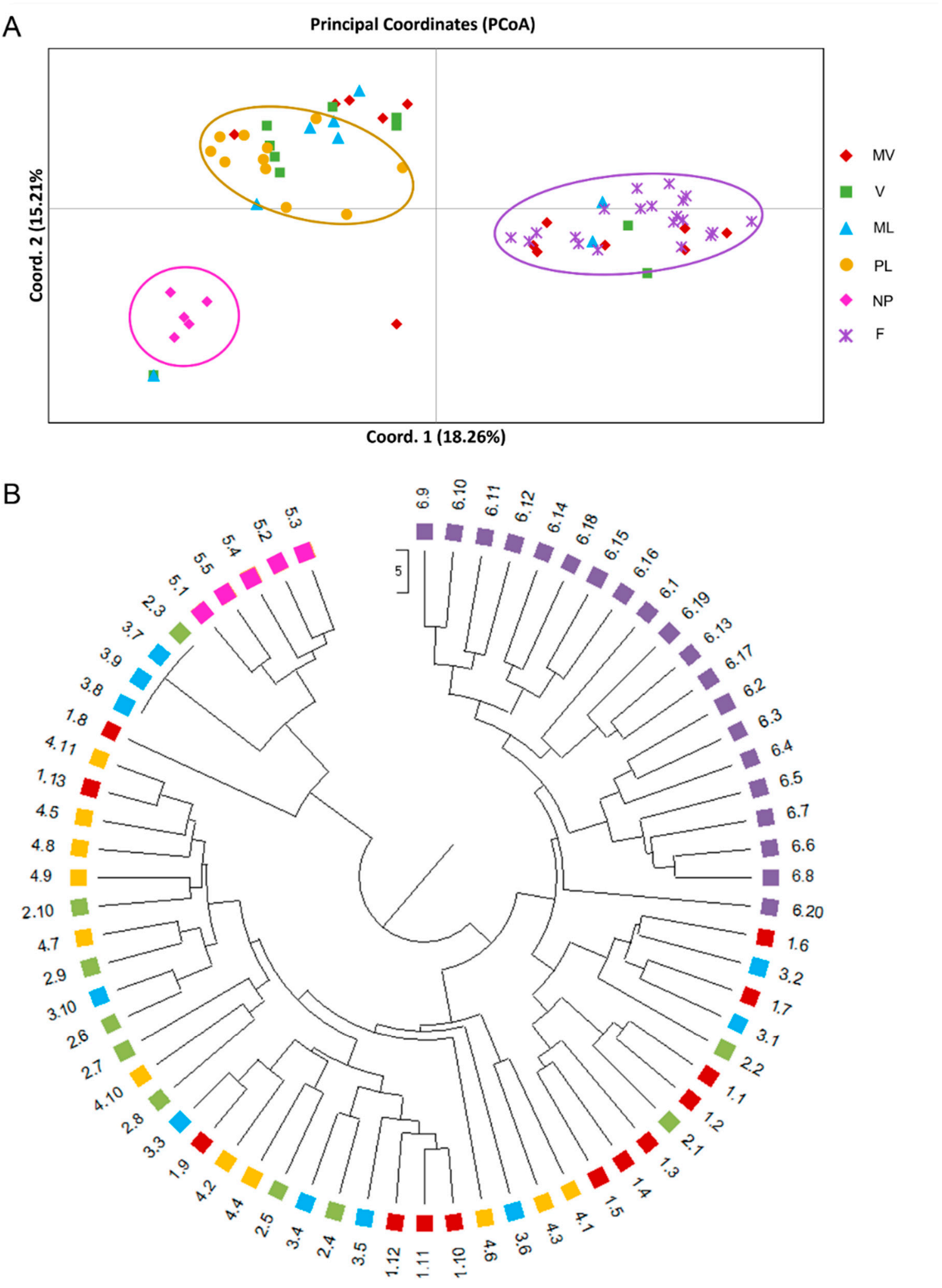

Figure S1. Genetic relationships of 20 female and 49 male individuals of five *P. lentiscus* var. *chia* cultivars based on the ISSR polymorphism analysis. A. Principal Coordinate Analysis (PCoA) based on 134 polymorphic ISSR loci. The first two coordinates accounted for 33.47% of the total variance (axis 1 = 18.26%; axis 2 = 15.21%). B**.** The unweighted pairgroup method using arithmetic average (UPGMA) dendrogram of the six populations.

Table S4. Transcriptome-based primers designed based on the markers selected.

#	Name	Sequence	Product size	Ta	Transcript annotation	Type
1	MYB1_F	AGTAGTGGTAGGAGTTCGGTGAT	100	50	MYB DNA-binding masttrinLocDN1068c0g1t5.p1 length: 796bp	SNP/EST-SSR
	MYB1_R	CCGGATCCAACGCCGT				
	MYB3_F	ATTGCGGAGCATGTTGGTA	100	50		
	MYB3_R	TGGGAACCTCAATACTTTCTAGTAGG				
2	SUF4_F	ATGCGAAAGCTGGAAGAGAGT	100	52	SUF4 masttrinLocDN36c0g1t2.p1 length: 412bp	
	SUF4_R	TCTTCTTCTTCTTCTCCATAGTGAGC				
3	SPL91_F	ATGCTAAGGCTTACTATTCTAGGC	100	52	SPL9 masttrinNODE_19933_length_2204cov_75.335523_g9274_i1.p1 length: 397bp	
	SPL91_R	CACTGTTGGCAAAACCTCTGC				
	SPL92_F	ACAGCTGTTAATCAATATCCAAACG	100	52		
	SPL92_R	TTGGTCCCAAACCAAGGTG				
4	ELF61_F	ACTTCTTCAACTATCTCATTGG	100	52	ELF6 masttrinR17271602.p1 GENE.masttrinR17271602 length: 1627bp	
	ELF61_R	CAAGCTTCCAACCCG				
	ELF62_F	ATAATCATCAGACACGACAAATCATCA	100	53		
	ELF62_R	GCTGCATTGATATGTCCACCAG				
	ELF63_F	ATGTCTCTGTGAATGAGGTCTC	100	52		
	ELF63_R	ACCTTGTCCTCCATCTCTGT				
	ELF65_F	AGGGCTCAGACCGCAA	100	52		
	ELF65_R	GGGTTCTGCAGTACTTCTGAAT				
	ELF66_F	CTTCTTCATCTAAAGACGAAAGAGAGAT	100	53		
	ELF66_R	TCCACTTCACTCTTTCTTTTTCTTCC				
5	Rpr21_F	ACTGTTGCCACAGCTAATATCG	100-106	55	Rpr2 masttrinNODE_53355_length_1162cov_20.313131_g28763_i0.p1 length: 309bp	EST-SSR
	Rpr21_R	TAGTTTCTCCACTCTCATACTGCC				
6	ACS1_F	ATTGAGGACCCTGCTGGT	100	55	ACS masttrinLocDN2609c0g1t2.p1 length: 774bp	Differential Expression
	ACS1_R	TCCTGATGTCAAGATTCTCTGAG				
	ACS2_F	GGTATTATAATAATTTCTGTTTACCCGGTCT	156-141	55		
	ACS2-3_R	ACCCGAAAAAGACAAGGAGGAT				
	ACS3_F	ATAAAGCTCCAGTTTTGGTCTCAACA	105-90	55		
	ACS2-3_R	ACCCGAAAAAGACAAGGAGGAT				

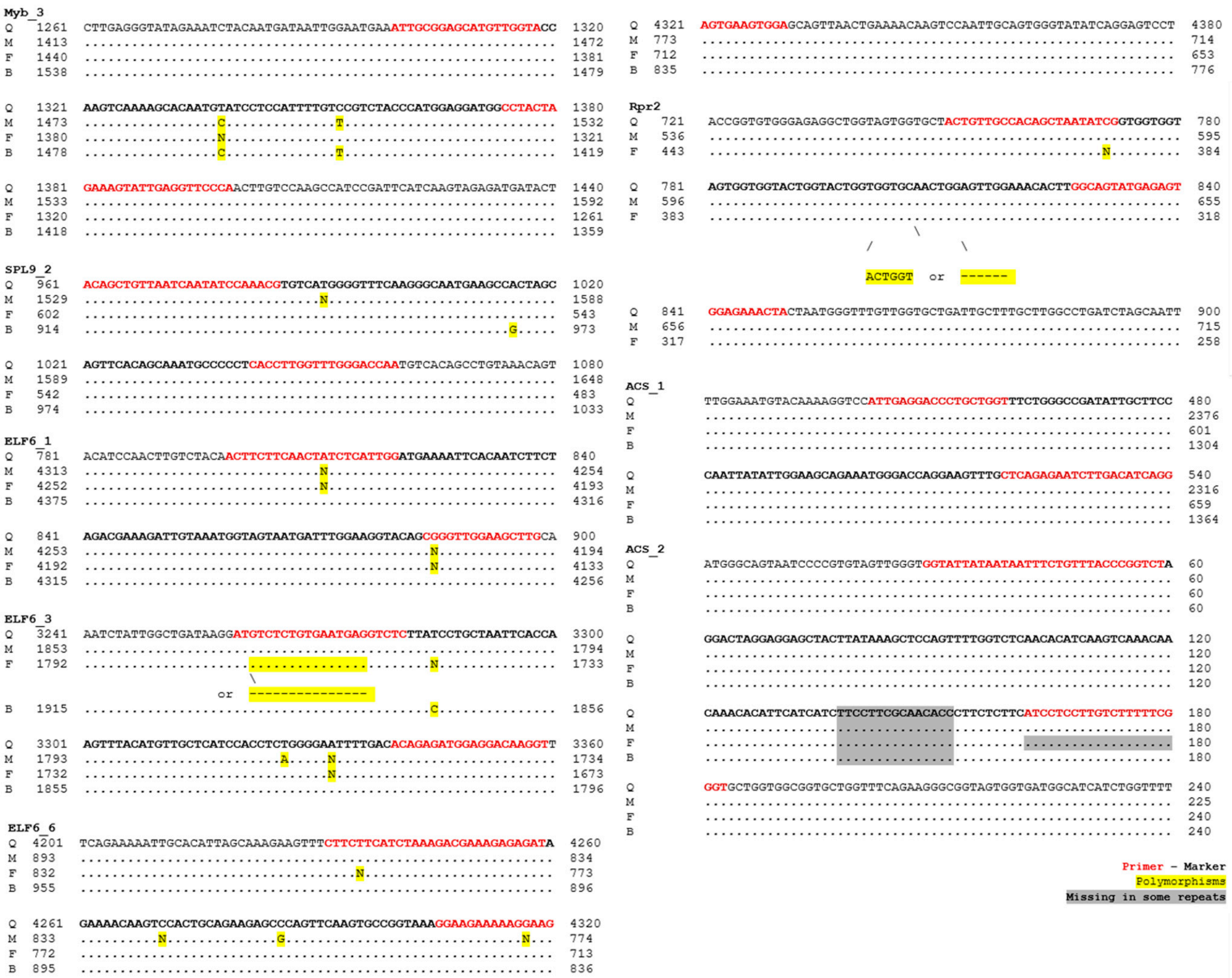

Figure S2. Primer design for the eight most informative markers based on the polymorphism information content (PIC) values (Table SX and Table 5) comprised of six EST-SSRs and two DEG-based markers (MYB3, SPL92, ELF61, ELF63, ELF66, Rpr21, ACS1 and ACS2) found in mastic trees. Letters stand for Q = Query, M = Male, F = Female, B = Monoecious. The product is presented with bold letters, while the primers used are shown in red. Polymorphisms are highlighted in yellow, while deletions found in some of the repeats are highlighted in grey.

Table S5: Average PIC and standard error (SE) values for the transcriptome-based markers used for distinguishing male and female mastic trees.

Marker	Average PIC value MALE	Average PIC value FEMALE	Average PIC value ALL
MYB1	0.472±0	0.437±0.0124	0.4612±0.0036
MYB3	0.454±0.0275	0.397±0.0159	0.4417±0.0175
SPL91	0.413±0.0606	0.4375±0.0124	0.4335±0.0349
SPL92	0.371±0.0312	0.475±0.0141	0.4667±0.0158
HYPA1	0.311±0.0734	0.235±0.099	0.2846±0.0824
ELF61	0.392±0.0459	0.375±0.0849	0.4571±0.0284
ELF62	0.447±0.0067	0.37±0.0354	0.4503±0.0347
ELF63	0.309±0.1046	0.235±0.099	0.3186±0.1283
ELF65	0.358±0.022	0.235±0.099	0.3182±0.0466
ELF66	0.284±0.1223	0.4375±0.0124	0.3885±0.0783
RpR21	0.344±0.0661	0.24±0.1697	0.345±0.0145
ACS1	0.278±0.156	0.3475±0.0194	0.3285±0.1135
ACS2	0.323±0.1144	0.3775±0.0866	0.3985±0.0233
ACS3	0.323±0.1144	0.435±0.0424	0.4009±0.039

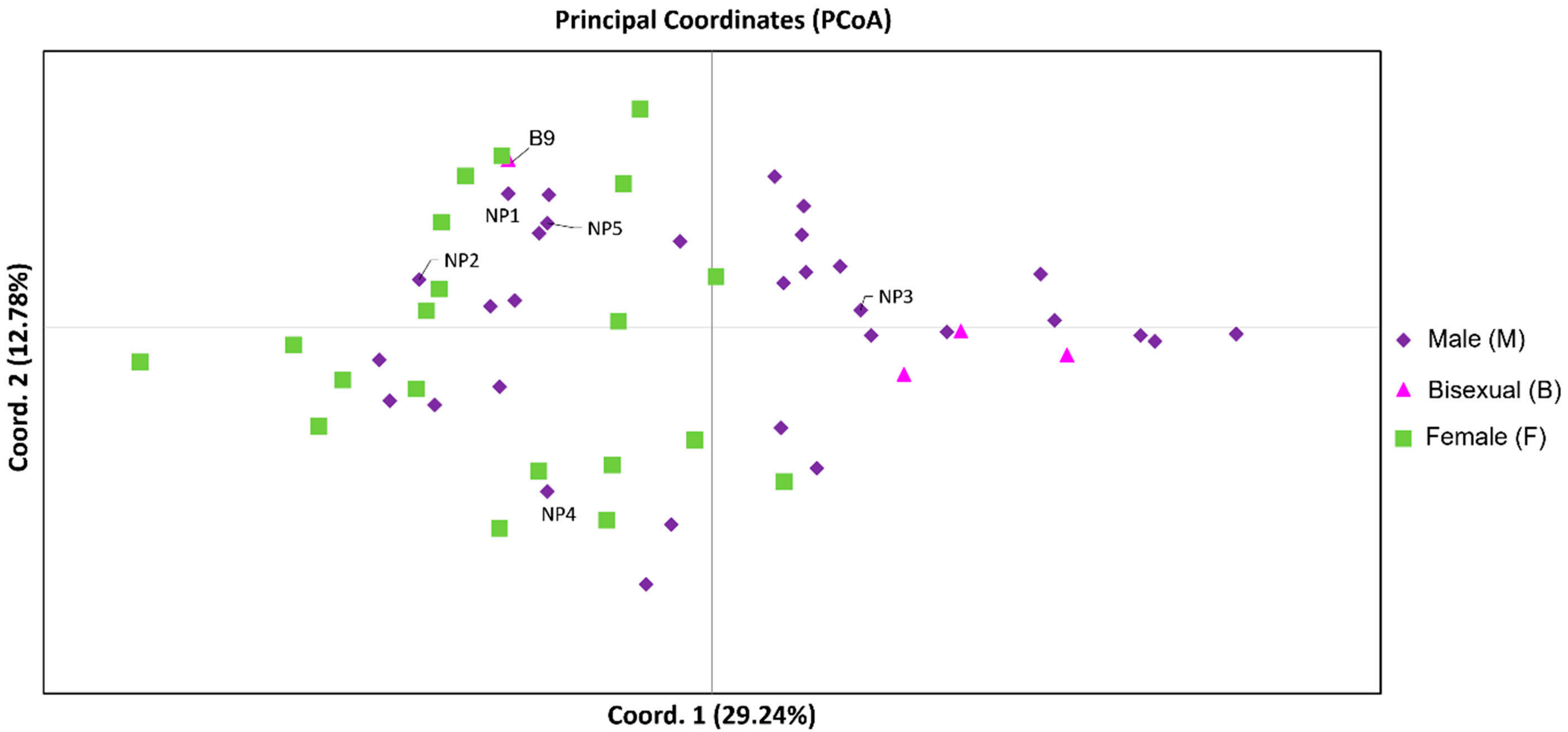

Figure S3. Principal Coordinate Analysis (PCoA) of the 34 male and 5 non-productive male individuals along with four bisexual and 20 female *P. lentiscus* var. Chia individuals based on the developed 14 ESTSSR and four DEG-based polymorphic loci. The first two coordinates accounted for 42.02% of the total variance (axis 1 = 29.24%; axis 2 = 12.67%). The PCoA analysis shows the partial separation of the two sex types (male *vs.* female) as distinct groups. Most of the male NP individuals are grouped with the F population, whereas most three out of four bisexual individuals are grouped with the male population.
